# Factors that influence the pancreatic and duodenal microbiome in patients undergoing pancreatic surgery

**DOI:** 10.1371/journal.pone.0278377

**Published:** 2022-12-16

**Authors:** Eline S. Zwart, Suzanne Jeleniewski, Lenka N. C. Boyd, Laura L. Meijer, Jisce R. Puik, Barbara M. Zonderhuis, Freek Daams, Andries E. Budding, Reina E. Mebius, Geert Kazemier

**Affiliations:** 1 Department of Surgery, Cancer Center Amsterdam, Amsterdam Universities Medical Centers, VU University, Amsterdam, The Netherlands; 2 Department of Molecular Cell Biology and Immunology, Amsterdam Universities Medical Centers, VU University, Amsterdam, The Netherlands; 3 InBiome, Amsterdam, The Netherlands; University of Florida, UNITED STATES

## Abstract

**Background/Objectives:**

This study examined the correlation between pancreatic microbiome and patients characteristics. Furthermore, we compared different duodenal materials to examine their reflection of the pancreatic microbiome.

**Methods:**

Patients undergoing pancreatic surgery were included in the study. Characteristics of those patients were prospectively registered and sterile pancreatic biopsies were collected during surgery. After completion of the resection, duodenal fluid, -tissue and -swab were collected. Bacterial DNA was extracted and analyzed with IS-pro assay.

**Results:**

Paired samples of 51 patients were available for evaluation, including pancreatic biopsies from all patients, 22 duodenal fluids, 21 duodenal swabs and 11 duodenal tissues. The pancreatic microbiome consisted mostly of Proteobacteria followed by Firmicutes, Actinobacteria, Fusobacteria and Verrucomicrobia (FAFV) and Bacteroidetes. On species level, *Enterococcus faecalis*, *Escherichia coli*, and *Enterobacter-Klebsiella* were most abundant. In pancreatic biopsies, the total bacterial load and Proteobacteria load were significantly higher in patients with biliary drainage (54618.0 vs 5623.5; 9119.0 vs 2067.1). Patients who used proton pump inhibitors had a significantly higher total bacterial load (115964.7 vs 8495.8), more FAFV (66862.9 vs 1890.1), more Proteobacteria (24245.9 vs 2951.4) and more Bacteroidetes (542.5 vs 25.8). The head of the pancreas contained significantly more bacteria (21193.4 vs 2096.8) and more FAFV (5225.7 vs 19.0) compared to the tail, regardless of biliary drainage. Furthermore, the microbiome of all duodenal materials showed a weak correlation with the pancreatic microbiome.

**Conclusion:**

Biliary drainage, use of proton pump inhibitors, and anatomic location of the pancreatic biopsy influence the pancreatic microbiome. Furthermore, the duodenal microbiome does not suffice as a surrogate for the pancreatic microbiome.

## Introduction

The gut microbiome has gained interest in recent years as one of the potential drivers in cancer development [1]. Particularly in patients suffering from colon carcinoma, the gut microbiome appears to play a pivotal role in carcinogenesis, partly through the advocated modulating effect of the microbiome on the immune system [2,3]. For example, *Fusobacterium nucleatum* can upregulate the nuclear factor-κB signaling pathway, which is involved in carcinogenesis [4]. Furthermore, the microbiome is known to have an influence on the effectiveness of immunotherapy and chemotherapy by influencing the expression of multiple enzymes that are required for myeloid immune cells to produce reactive oxygen species, which are necessary for chemotherapy activity [5,6]. In pancreatic ductal adenocarcinoma (PDAC) however, less is known regarding the influence of the microbiome, consisting mostly of Proteobacteria, and especially the Enterobacteriaceae and Pseudomonadaceae families, on disease development or treatment [7]. Some studies have noted a correlation between the composition of the oral microbiome and the risk of PDAC development, but a causal link has not yet been described [8–10]. Particularly periodontal disease with a pathogenic oral microbiota, with increased abundance of *Porphyromonas gingivalis* and *Aggregatibacter actinomycetemcomitans*, was shown to be associated with an increased risk of PDAC development, whereas the genera *Fusobacterium* and *Leptotrichia* were associated with a decreased risk [11,12].

The route that the bacteria would have to follow to reach the pancreas from the mouth has been suggested to be either via the gastro-intestinal tract or via the bloodstream [13,14]. Pushalkar et al. showed that orally administrated fluorescently labeled bacteria in mice could reach the pancreas via the gastro-intestinal tract [14]. In humans however, this route of dissemination has not yet been confirmed. Furthermore, the occurrence of bacteria in the pancreas has not been shown to contribute to carcinogenesis in humans. In germ-free mice, there appears to be a protective effect caused by the lack of bacteria, leading to reduced pancreatic dysplasia and diminished intra-tumoral fibrosis in a spontaneous PDAC mouse model [14]. In addition, bacterial ablation in non-germ free mice also led to reduced tumor burden [14].

Even though various studies have documented the presence of bacteria in the human pancreas, these data have to be looked at with caution as patients suffering from tumors that involve the periampullary region often require preoperative biliary drainage to relieve biliary obstruction and jaundice [15]. This drainage procedure could contribute to translocation of bacteria from the duodenum towards the pancreas. By comparing patients with and without a biliary drainage, more insight could be gained into the baseline situation of the pancreatic microbiome and the possible change in bacterial load due to biliary drainage. At the time of biliary drainage, cytology is often obtained to determine a diagnosis by brushing the bile duct. Another possibility to confirm the diagnosis in those patients is by fine-needle aspiration biopsy guided by endoscopic ultrasound (EUS). These procedures could allow for acquisition of accurate information on the patient’s microbiome before further treatment but could also introduce bacteria into the pancreas leading to an altered microbiome profile after acquisition. If bacteria translocate via the gastro-intestinal tract, the duodenal microbiome might be an adequate reflection of the pancreatic microbiome and could be obtained and examined more easily compared to pancreatic tissue.

Therefore, this study aimed to explore the perioperative pancreatic microbiome in patients with and without a biliary drainage in order to investigate the possible contribution of biliary drainage to the local microbiome. Furthermore, we set out to examine the correlation between the microbiome and other patient characteristics. Finally, to assess if the pancreatic microbiome can be mimicked without obtaining pancreatic tissue, we compared different duodenal materials to see if this would be an adequate reflection of the pancreatic microbiome to suffice as a surrogate.

## Methods

### Study design

The study design and protocol were approved by the local Medical Ethics Board of the Amsterdam UMC, VU University Amsterdam (#2016.510) in accordance with the ethical guidelines of the Declaration of Helsinki. Before study participation, written informed consent was obtained from all participants. Patients undergoing pancreatic surgery between August 2018 and September 2020 for various indications were included in the study. Final diagnosis was based on histopathological examination of the resected specimen by the pathologist. Clinicopathological characteristics and patient outcomes were collected in a prospective database. The nutritional status was based on the SNAQ score, calculated by a dietician at the first outpatient visit. A score of ≥3 was defined as poor nutritional status [16]. Survival was calculated from the date of surgery until the date of last follow up or death. Overall survival (OS) was categorized as short survival for patients who died within one year after surgery, and long survival as survival longer than one year after surgery. All patients who underwent a pancreatic resection less than one year ago and were still alive, were excluded from the survival analysis.

### Biliary drainage procedures and invasive preoperative diagnostic procedures

Patients requiring preoperative biliary drainage to relieve biliary obstruction, underwent endoscopic retrograde cholangiopancreatography (ERCP) with stent placement, percutaneous transhepatic cholangiodrainage (PTCD) catheter, or both. Only prior to PTCD placement, 2000 mg ceftriaxone was given intravenously. For the ERCP with stent placement, the gastroenterologist positioned a gastroscope via the oral cavity in front of the ampulla of Vater. Here an papillasphicterotomy was performed, followed by the introduction of a plastic or self-expandable metal stent (SEMS) protruding from the duodenum into the common bile duct and additionally, if cytology was requested, a brush cytology of the distal bile duct was performed.

In case of a PTCD, a radiologist inserted a catheter transpercutanously and transhepaticallyinto the bile duct towards the papilla of Vater into the duodenum. The external part of the drain was closed in the case that adequate drainage towards the duodenum was attained.

For a diagnostic pancreatic biopsy, the gastroenterologist inserted the gastroscope via the oral cavity and placed it in the duodenum. Guided by EUS, a fine needle biopsy or aspiration was acquired from the pancreatic lesion through the duodenal wall.

### Sample processing

Thirty minutes prior to the operation, all patients received standard antibiotic prophylaxis consisting of 2000 mg cefazolin and 500 mg metronidazole intravenously, followed by intraoperative antibiotics every 4 hours for the duration of the operation. After a subcostal incision or laparoscopic introduction, and after mobilization of the pancreatic head and duodenum through a Kocher’s maneuver for periampullary tumors or the pancreatic body and/or tail when applicable, a sterile needle biopsy of non-fibrotic pancreatic tissue was taken by the surgeon directly after resectability of the specimen was confirmed. After completion of the resection, duodenal fluid was collected at the pathology department when applicable in patients that underwent a pancreatoduodenectomy. To prevent contamination of the samples taken from resected material, before opening of the duodenum, duodenal fluid was aspirated with a sterile needle through the duodenal wall and collected in a 2 mL Eppendorf vial. Directly after opening of the duodenum in those patients, a swab (eSwab 480CE, Copan) was taken from the papilla of Vater or in proximity in case of a biliary stent or PTCD catheter in-situ and stored in the provided corresponding tube with Liquid Amies Medium. Finally, a full-thickness part of healthy duodenal tissue was collected in patients that underwent a pancreatoduodenectomy. All samples were snap-frozen in liquid nitrogen and stored at -80°C until further processing.

### DNA isolation

For DNA extraction, three protocols were employed. For duodenal tissue and pancreatic tissue, a piece of 3x3 mm was cut and added to an Eppendorf tube with 500 μl IBX-buffer (inBiome, Amsterdam, The Netherlands) and 400 mg Zirconia / Silica beads, 0.1mm (Biospec, 11079101Z). Bead beating was performed for 180 seconds at room temperature. After bead beating, tubes were centrifuged, and supernatant was transferred to new containers. The supernatant was vortexed and incubated at 95˚C while shaking at 800 rpm for 10 minutes. Thereafter, 50 μl of 1 M Tris / HCl pH7.0 (Fisher scientific, 10274773) was added and tubes were centrifuged shortly. The complete mixture was added to an easyMAG container, together with 1 ml of lysis buffer (Biomérieux) and 1 ml AL buffer (Qiagen, 19075). For duodenal fluid DNA extraction, 1 ml of duodenal fluid was added to an easyMAG container, together with 2 ml of lysis buffer (Biomérieux). For DNA isolation from the swabs, 1 ml of lysis buffer (Biomérieux) was added to each tube containing a swab tip and the mixture was shaken at 1400 rpm for five minutes. Afterwards, all samples were centrifuged for two minutes at 14.000 rpm. 2 ml of supernatant was added to an easyMAG container, together with 1 ml of lysis buffer (Biomérieux). All mixtures from the three different protocols were incubated for at least 10 minutes, before adding 70 μl of Magnetic Silica (Biomérieux). DNA extraction was performed on the NucliSENS easyMAG automated DNA isolation machine (Biomérieux) with the specific A protocol, as described by the manufacturer. The DNA was eluted in 70 μl buffer and stored at 4°C prior to IS-profiling.

### IS-profiling

Microbiota analysis was performed by Molecular Culture, a 16S-23S ribosomal DNA (rDNA) based bacterial profiling technique, optimized for clinical use. This procedure was performed following the manufacturer’s instructions for use (inBiome, Amsterdam) [17]. For each run, a positive and negative control were included and a the same input volume was used. In the Molecular Culture technique, bacterial DNA is amplified with three different primer sets for different phylum groups: Proteobacteria, Bacteroidetes and FAFV, where FAFV is a combination of the phyla Firmicutes, Actinobacteria, Fusobacteria and Verrucomicrobia. Bacterial taxa can generally be resolved to the species level by matching to a curated, in proprietary database of inBiome. Bacterial loads are expressed in Relative Fluorescence Units (RFU), which is a corollary of abundance of bacteria.

### Statistical analysis

Sample data was processed in and extracted from TIBCO Spotfire and statistical analysis subsequently was performed in SPSS, version 26 [18,19]. The relative abundance was calculated per sample and bacterial loads were expressed as median with an interquartile range. Continuous variables were analyzed with a Student *t* test or Mann-Whitney U test as appropriate, categorical variables were analyzed with a Pearson Chi Square test. Paired samples were analyzed using the related-Samples Wilcoxon Signed Rank Test. The Shannon diversity index and cosine correlation were calculated in TIBCO Spotfire and subsequently also analyzed in SPSS. For phyla specific analysis, only species or abundance of the designated phyla was included in the analysis. A p value of ≤ 0.05 was considered statistically significant. Data were visualized in SAS Visual analytics platform (SAS Institute Inc, Cary, North Caroline, USA).

## Results

### Patient characteristics

A total of 51 patients were included, with a mean age of 67.0 years, and 30 patients were male. Twenty nine patients (56.9%) had a poor nutritional status, requiring high protein drink supplements in 19 patients and feeding via a tube in 7 patients. Thirty-three patients (64.7%) underwent biliary drainage prior to operation. Of those, 29 were performed by an ERCP through the papilla and four patients received a PTCD catheter that was placed trans percutaneously through the papilla into the duodenum. Furthermore, from 31 patients (60.8%) a preoperative biopsy was taken trans duodenally under EUS guidance to confirm the diagnosis. Forty-one patients (80.4%) underwent a pancreatoduodenectomy, seven (13.7%) underwent a pancreatic tail resection, one (2.0%) underwent a Frey’s procedure, and one (2.0%) duodenum preserving right sided pancreatectomy and one (2.0%) gastro-enteral bypass were performed. Most of the patients had a malignant tumor, of which PDAC was the most common histopathological diagnosis (Table 1). Of all patients, an intraoperatively obtained pancreatic needle biopsy was available for analysis. Forty-four (86.3%) were taken at the head of the pancreas and seven (13.7%) at the body and/or tail of the pancreas. Furthermore, matched duodenal material was available of 24 patients, consisting of 22 duodenal fluids, 21 swabs and 11 duodenal tissues.

**Table 1:** Patient characteristics.

	Patients (n = 51)
Age, mean (SD)	67.0 (10.2)
Sex (M/F), n (%)	30/21 (58.8/41.2)
Proton pump inhibitor users, n (%)	20 (39.2)
Diabetes mellitus n (%)	15 (29.4)
BMI n (%)	
<25	29 (56.9)
25–30	16 (31.4)
>30	6 (11.8)
Biliary drainage, n (%)	
Yes	33 (64.7)
Internal–Metal stent	22 (66.7)
Internal–Plastic stent	7 (21.2)
External—Percutaneous transhepatic cholangiodrainage catheter	4 (12.1)
No	18 (35.3)
EUS, n (%)	31 (60.8)
Neoadjuvant therapy, n (%)	8 (15.7)
Histology, n (%)	
Malignant	41 (80.4)
PDAC	27 (52.9)
Papilla carcinoma	6 (11.8)
Duodenal carcinoma	3 (5.9)
Cholangiocarcinoma	3 (5.9)
Metastasis RCC	1 (2.0)
Benign	10 (19.6)
Chronic pancreatitis	5 (9.8)
Fibrosis	2 (3.9)
Sereus cyst adenoma	1 (2.0)
Neuroendocrine tumor	1 (2.0)
IPMN	1 (2.0)
Healthy	1 (2.0)
Location of pancreatic biopsy, n (%)	
Head	44 (86.3)
Tail	7 (13.7)
Complications, n (%)	36 (70.6)
Adjuvant therapy, n (%)	14 (27.5)

Percentages are denoted for the above-mentioned group. Abbreviations: BMI: Body Mass Index, EUS: Endoscopic Ultrasound, PDAC: Pancreatic ductal adenocarcinoma, RCC: Renal cell carcinoma, IPMN: Intraductal papillary mucinous neoplasm.

### Pancreatic microbiome in pancreatic tissue

**Fig 1:**
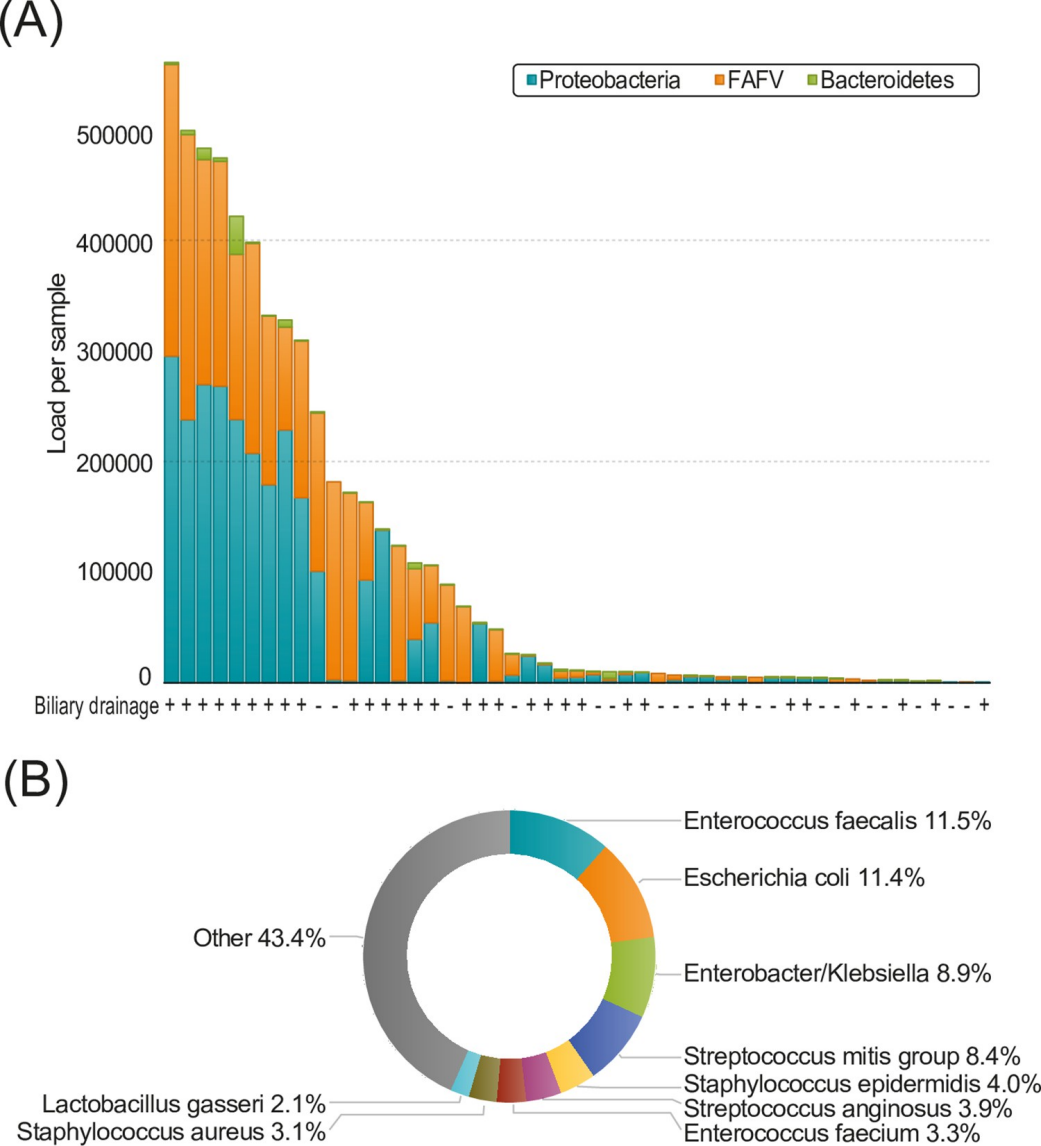
**(A)** Total load per phylum in pancreatic biopsy samples (n = 51). The + indicates that the patient underwent biliary drainage, the–indicates that the patient did not undergo biliary drainage. **(B)** Species present in pancreatic biopsy samples. Percentages are calculated over the total amount of bacteria.

**Fig 2:**
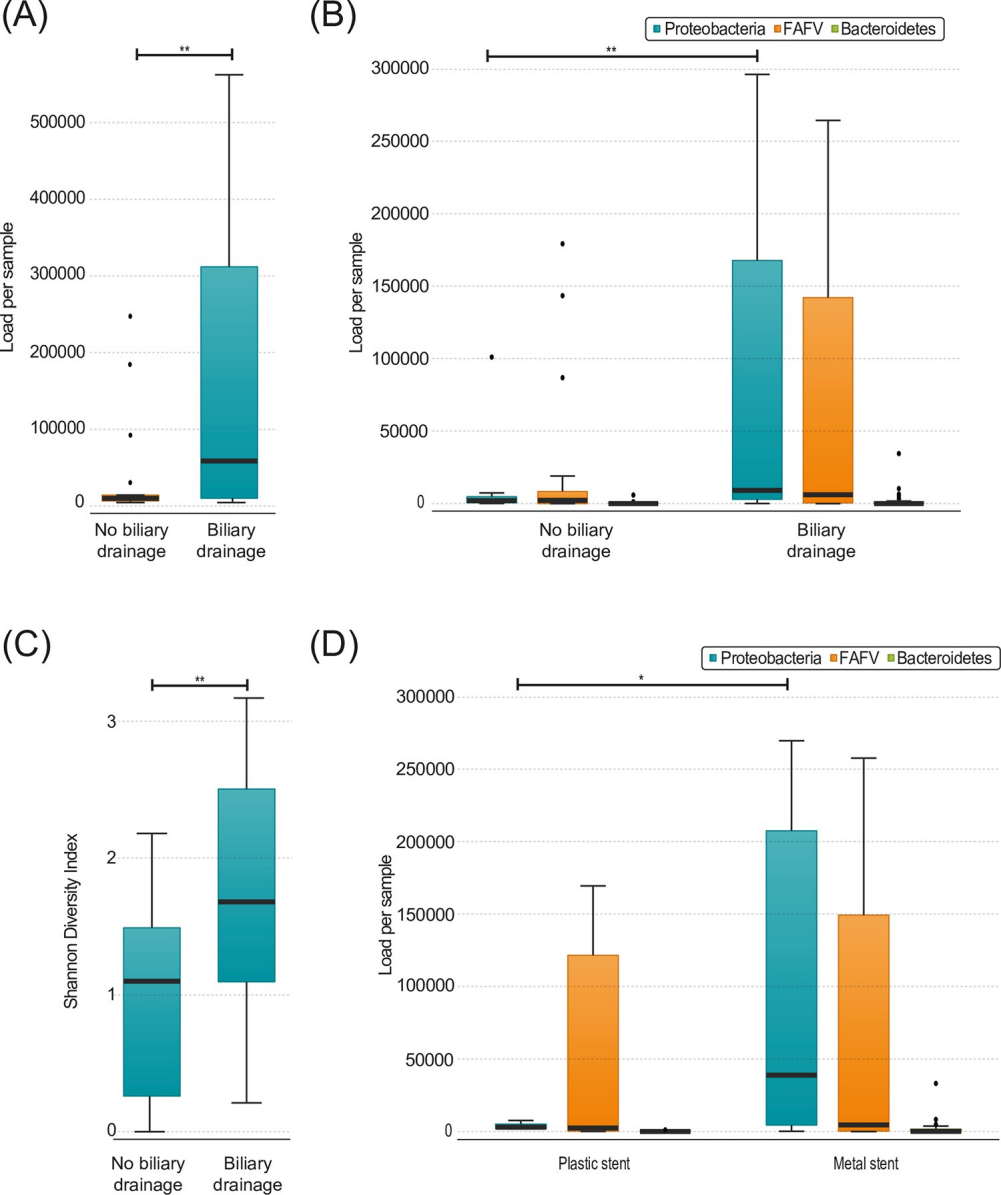
**(A)** Total load in pancreatic biopsies, divided by patients with (n = 33) and without (n = 18) biliary drainage (p = 0.007) **(B)** Load per phylum in pancreatic biopsies, divided by patients with (n = 33) and without (n = 18) biliary drainage (Proteobacteria p = 0.003) **(C)** Shannon diversity index of pancreatic biopsies, divided by patients (n = 33) and without (n = 18) biliary drainage (p = 0.014) **(D)** Load per phylum in pancreatic biopsies, divided by patients with a plastic (n = 7) and a metal stent (n = 22) in the distal bile duct (Proteobacteria p = 0.032). Boxplots: Black bar denotes median, box denotes the interquartile range, whiskers indicate the range of values that are outside of the interquartile range. Outliers are defined as >1.5 times the size of the interquartile range and presented as a black dot.

To determine the presence of various microbial strains in the obtained samples, bacterial profiling was performed on all samples collected from the pancreas. Most commonly, Proteobacteria were found in the pancreas tissue, followed by FAFV and Bacteroidetes, respectively (Fig 1A). Specifically, the microbiome consisted mostly of *Escherichia coli*, *Enterobacter-Klebsiella*, *Enterococcus faecalis and Steptococcus mitis* group (Fig 1B). The median bacterial load was significantly higher in patients that underwent biliary drainage compared to patients that did not undergo biliary drainage (54618.0 vs 5623.5, p = 0.007; Fig 2A). Furthermore, a significantly higher Proteobacteria load was observed in patients that underwent biliary drainage (9119.0 vs 2067.1, p = 0.003; Fig 2B. For both FAFV and Bacteroidetes, there was a clear trend towards a higher load in patients that underwent biliary drainage (6093.8 vs 2234.2 p = 0.070, 59.2 vs 27.6, p = 0.070 respectively), but it did not reach statistical significance (Fig 2B). In addition, patients that underwent biliary drainage showed a higher diversity (2.55 vs 1.74, p = 0.014; Fig 2C) and a higher Proteobacteria specific diversity (1.79 vs 0.73, p = 0.042). No difference in bacterial load was observed between patients with an internal stent or PTCD catheter. Patients who received a SEMS had significantly more Proteobacteria compared to patients with a plastic stent or PTCD catheter (38826.0 vs 2951.4, p = 0.032; Fig 2D).

**Fig 3:**
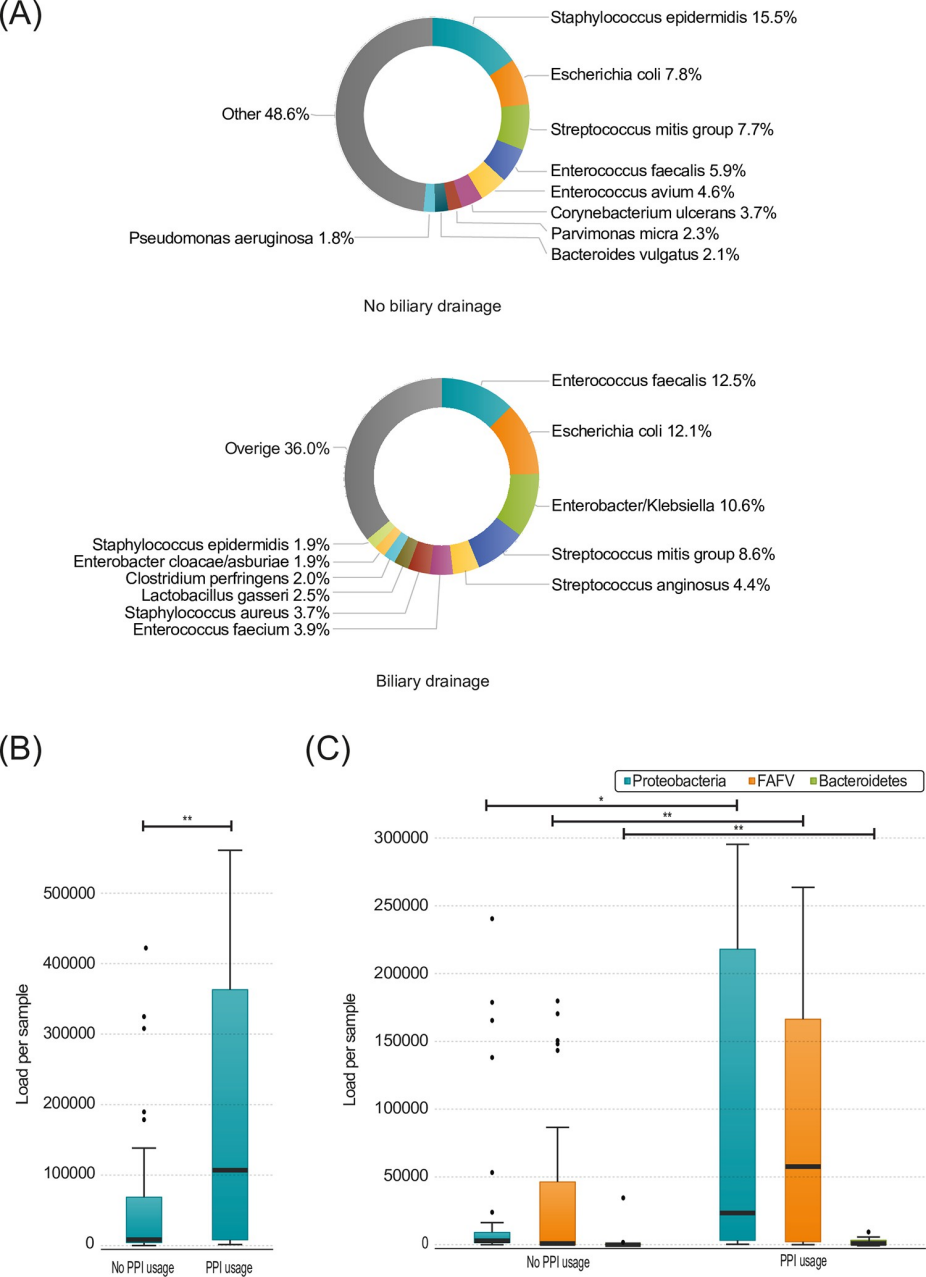
**(A)** Species present in pancreatic biopsy samples with and without biliary drainage. Percentages are calculated over the total amount of bacteria. **(B)** Total load in pancreatic biopsies, divided by patients (n = 20) and without (n = 31) PPI usage (p = 0.007) **(C)** Load per phylum in pancreatic biopsies, divided by patients (n = 20) and without (n = 31) PPI usage (FAFV p = 0.008). Boxplots: Black bar denotes median, box denotes the interquartile range, whiskers indicate the range of values that are outside of the interquartile range. Outliers are defined as >1.5 times the size of the interquartile range and presented as a black dot. Abbreviations: PPI: Proton pump inhibitor.

At a species level, in patients that received a biliary drainage procedure, there was a relatively higher abundance of *Enterococcus faecalis* (12.5% vs 5.9%), *Escherichia coli* (12.1% vs 7.8%) and *Enterobacter/Klebsiella* (10.6% vs <0.1%). In contrast, there were less *Staphylococcus epidermidis* (1.9% vs 15.5%) in those patients (Fig 3A). In summary, biliary drainage affects the microbial load and diversity of the microbiome within the pancreatic tissue.

In addition, patients that used proton pump inhibitors (PPI) had a higher bacterial load in the pancreatic tissue (115964.7 vs 8495.8, p = 0.007; Fig 3B). Furthermore, the microbiome contained more FAFV (66862.9 vs 1890.1, p = 0.008), Proteobacteria (24245.9 vs 2951.4, p = 0.015) and Bacteroidetes (542.5 vs 25.8, p = 0.007; Fig 3C).

### Pancreatic carcinoma

**Fig 4:**
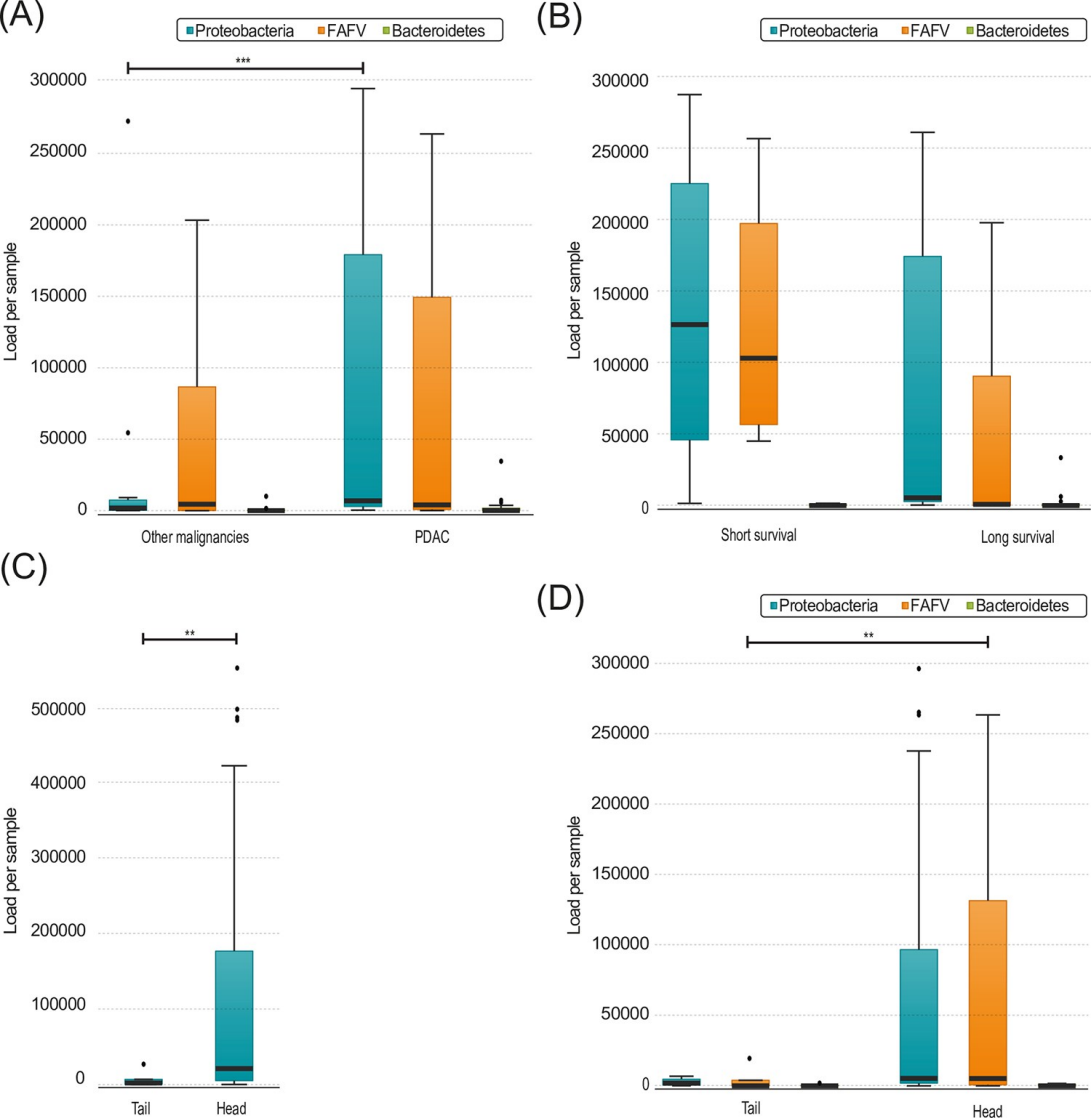
**(A)** Load per phylum in pancreatic biopsies, divided by patients with PDAC (n = 27) and other malignancies (n = 14) (Proteobacteria p = 0.027) **(B)** Load per phylum in pancreatic biopsies of patients with PDAC, divided by patients with short (n = 4) and long survival (n = 15) (FAFV p = 0.062) **(C)** Total load in pancreatic biopsies, divided by head (n = 44) and tail (n = 7) biopsies (p = p = 0.004) **(D)** Load per phylum in pancreatic biopsies, divided by head (n = 44) and tail (n = 7) biopsies (FAFV p = 0.004, Proteobacteria p = 0.073). Boxplots: Black bar denotes median, box denotes the interquartile range, whiskers indicate the range of values that are outside of the interquartile range. Outliers are defined as >1.5 times the size of the interquartile range and presented as a black dot. Abbreviations: PDAC: Pancreatic ductal adenocarcinoma.

Patients with PDAC had more Proteobacteria in the pancreatic microbiome compared to other malignancies (7580.6 vs 1872.6, p = 0.001; Fig 4A), however this difference disappeared in the subgroup analysis including only patients with a biliary drainage as only 6 patients with a different type of malignancy could be included for these analysis. There was no difference in bacterial load between patients with malignancies compared to patients suffering from benign diseases. Patients with malignancies who received neoadjuvant treatment have more Proteobacteria (38826.0 vs 4160.2, p = 0.038) and more Bacteroidetes (644.5 vs 30.2, p = 0.038). Subgroup analysis of only patients with PDAC or patients that underwent biliary drainage, demonstrated no significant differences between the bacterial load.

There was no significant difference in total bacterial load comparing patients with PDAC with short and long OS, nor per phylum. However, it was observed that a higher load of FAFV correlated with a tendency towards shorter OS (105920.9 vs 923.4, p = 0.062; Fig 4B). Subgroup analysis of patients that underwent biliary drainage showed this same trend, and appeared thus independent of biliary drainage. Furthermore, there was no difference in the Shannon diversity Index between patients with short or long OS. Due to the high number of different species and limited patients with sufficient survival data, no specific species could be correlated to better survival. In summary, patients with PDAC displayed more Proteobacteria compared to other malignancies and a trend towards a better survival was found in these pancreatic tissue samples with a lower abundance of FAFV.

### Head versus tail biopsies

To see if the bacteria are more likely to enter the pancreatic tissue via the bloodstream or via the gastro-enteral tract, we compared the bacterial load between the head and the tail of the pancreas. The head of the pancreas contained significantly more bacteria compared to the tail (21193.4 vs 2096.8, p = 0.004; Fig 4C). There were significantly more FAFV (5225.7 vs 19.0, p = 0.004) and a trend towards more Proteobacteria in the pancreatic head compared to the tail (5357.5 vs 2023.4, p = 0.073; Fig 4D). Both total bacterial load and FAFV remained significantly increased in the head of pancreas in patients that did not undergo biliary drainage, suggesting that bacterial translocation from the duodenum is also possible without biliary drainage. Furthermore, the higher microbial load in the head of the pancreas is suggestive for translocation from the gastro-enteral tract rather than via the bloodstream or lymphatic tract.

Finally, sex, diabetes mellitus, preoperative EUS, malignant versus benign disease, postoperative complications or adjuvant therapy did not influence bacterial load or Shannon diversity index. For all comparisons of the bacterial load, see S2 File.

### Comparability of pancreatic and duodenal microbiome

**Fig 5:**
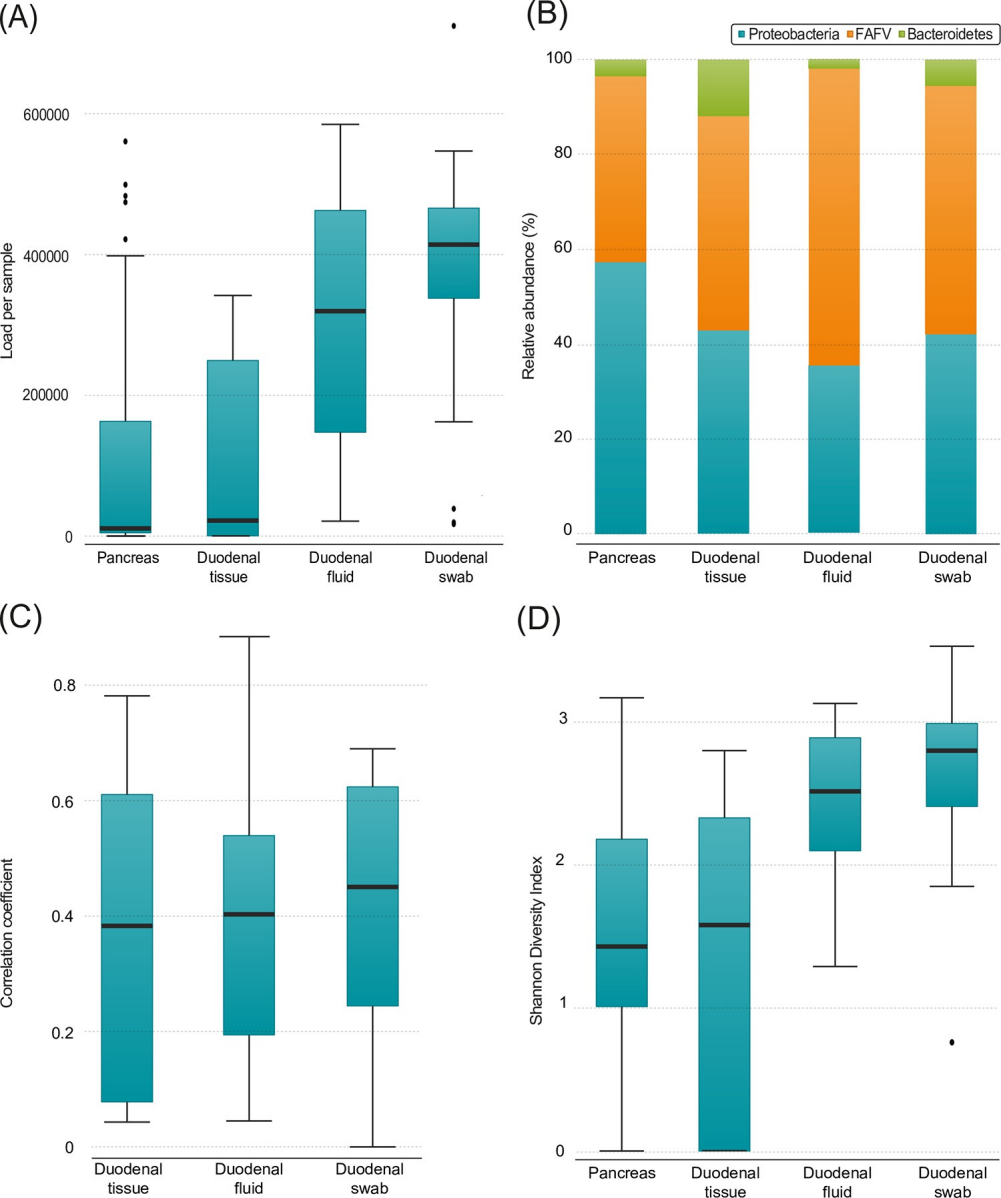
**(A)** Total load per sample (duodenal swab (n = 21) vs pancreatic tissue (n = 51) p = <0.001, duodenal swab (n = 21) vs duodenal tissue (n = 11) p = 0.002, pancreatic tissue (n = 51) vs duodenal fluid (n = 22) p<0.001, duodenal tissue (n = 11) vs duodenal fluid p = 0.014) (**B)** Relative abundance of phyla per sample. Pancreatic tissue (n = 51), duodenal tissue (n = 11), duodenal fluid (n = 22), duodenal swab (n = 21) (**C)** Correlation coefficient between the pancreatic biopsies and denoted material within the same patient. Duodenal tissue (n = 11), duodenal fluid (n = 22), duodenal swab (n = 21) **(D)** Shannon diversity index per sample (duodenal swab (n = 21) vs duodenal tissue (n = 11) p = 0.004, duodenal swab (n = 21) vs pancreatic tissue (n = 49) p<0.001, duodenal fluid (n = 22) vs duodenal tissue (n = 11) p = 0.043, duodenal fluid vs pancreatic tissue (n = 49) p = 0.001). Boxplots: Black bar denotes median, box denotes the interquartile range, whiskers indicate the range of values that are outside of the interquartile range. Outliers are defined as >1.5 times the size of the interquartile range and presented as a black dot.

Upon analyses of the different duodenal samples, the highest bacterial load was found in the duodenal swab, which was significantly higher compared to pancreatic tissue (414219.38 vs 10910.64, p<0.001) and duodenal tissue (414219.38 vs 98919.00 p = 0.002). There was also a significantly higher load in duodenal fluid compared to pancreatic tissue (319654.84 vs 10910.64, p<0.001) and duodenal tissue (319654.84 vs 98919.00, p = 0.014; Fig 5A). In a paired analysis, the bacterial load of duodenal fluid and swab remained significantly higher compared to the pancreatic tissue (414219.38 vs 54617,98, p = 0.001 and 319654.84 vs 51319,54, p <0.001). In addition, comparable to the pancreatic microbiome, patients that used PPI had more FAFV (212948.6 vs 157089.4, p = 0.018) and a trend towards a higher bacterial load of the duodenal swab (453175.1 vs 348744.0, p = 0.082), suggesting that these bacteria can survive due to less acidic stomach and duodenal fluid.

The highest mean relative abundance of Proteobacteria was found in pancreatic tissue. In addition, there were significantly more Proteobacteria in pancreatic tissue compared to duodenal fluid (57.3% vs 35.3%, p = 0.004). The highest mean degree of FAFV was to be found in the duodenal fluid and this was also significantly more than in pancreatic tissue (62.8% vs 39.2%, p = 0.001; Fig 5B) and remained borderline significant in the paired subgroup analysis, suggesting a selection of bacteria that are able to survive within pancreatic tissue. The relative abundance for the paired samples is depicted in S1 Fig.

To address the question whether the pancreatic microbiome could be derived from the microbiome present within the duodenum, the composition of both locations was compared. There was a weak correlation between the pancreatic microbiome and the duodenal tissue, duodenal fluid, and duodenal swab (R = 0.36, R = 0.39 and R = 0.41 respectively; Fig 5C). Biliary drainage did increase the correlation between the pancreas and duodenal tissue, fluid, and swab slightly (R = 0.39, R = 0.44 and R = 0.46). Between the different duodenal samples themselves, there was a higher, but still weak to moderate correlation. Between the duodenal fluid and swab, the correlation was R = 0.66 and in patients with biliary drainage 0.71. Between duodenal fluid and the duodenal tissue, R = 0.46 and R = 0.53 in patients with biliary drainage. The duodenal tissue and duodenal swab had the lowest correlation of R = 0.39 and R = 0.52 in patients with biliary drainage. The weak to moderate correlation might suggest that a specific micro-milieu is optimal for outgrowth of different bacterial species.

The composition of the microbiome had the highest diversity within the duodenal swab samples, with a median Shannon Diversity Index of 2.84, which was more diverse compared to duodenal tissue (2.84 vs 2.37, p = 0.004), and pancreatic biopsy (2.84 vs 2.40, p<0.001). Duodenal fluid also showed more diverse composition compared to duodenal tissue (2.67 vs 2.37, p = 0.043) and the pancreatic biopsy (2.67 vs 2.40, p = 0.001; Fig 5D). In contrast, in the paired samples, only the duodenal tissue had a significant difference with the pancreatic biopsy (2.33 vs 1.90, p = 0.002). The differences were mostly caused by significant differences in the FAFV phyla (duodenal swab 2.20 vs duodenal tissue 1.47, p = 0.005; duodenal swab 2.20 vs pancreatic biopsy 1.74, p = 0.001; duodenal fluid 2.06 vs duodenal tissue 1.47, p = 0.017; duodenal fluid 2.06 vs pancreatic biopsy 1.74, p = 0.007) and partly by to Proteobacteria (duodenal swab 2.03 vs pancreatic biopsy 1.67, p = 0.006; duodenal fluid 1.76 vs pancreatic biopsy 1.67, p = 0.004). In the paired samples, the FAFV was also significantly different between all duodenal materials and the pancreatic biopsy (duodenal swab 2.17 vs pancreatic biopsy1.04, p = 0.032; duodenal fluid 1.91 vs pancreatic biopsy 1.15, p = 0.035; duodenal tissue 1.71 pancreatic biopsy 0,71, p = 0.05. For the complete overview of the paired analysis, see Appendix I. The lower diversity of the pancreatic tissue also supports the hypothesis that a selection of bacteria takes place at the level of which bacteria that translocate towards and survive within the pancreatic tissue.

## Discussion

This is the first explorative study that shows that biliary drainage correlates with an increased bacterial load in the pancreas. Furthermore, various factors influencing the pancreatic microbiome were found, including the use of PPIs, the type of pancreatic malignancy and the anatomical location within the pancreas. Finally, this study is the first to show that the duodenal microbiome does not suffice as surrogate for the characterization of the pancreatic microbiome.

Approximately half of the patients operated for periampullary malignancies undergo biliary drainage preoperatively [15]. Therefore, it is pivotal to understand its effect on the duodenal and pancreatic microbiome. This is the first study to show that biliary drainage results in the increase in microbial load and diversity, and to show that without biliary drainage, only a limited microbial load is present. A previous study by Geller et al., studying the presence of bacteria in PDAC tissue by real-time quantitative polymerase chain reaction (PCR) of 16S rDNA in 113 patients and microbiome composition by deep sequencing of PCR-amplified bacterial 16S rDNA in 65 patients, did mention whether patients underwent an ERCP procedure before surgery, but not whether a stent or PTCD catheter was placed [7]. Unfortunately, it is not always possible to insert a stent during an ERCP procedure, as it may be too difficult to bypass the tumor and allow for the stent to be put into place. Therefore, only data regarding the ERCP and not the actual stent or PTCD catheter placement is too limited to investigate the effect of biliary drainage on the microbiome composition and bacterial load. Furthermore, data regarding the ERCP was missing for 62% of the patients [7].

The increase in microbial load and diversity seen in patients with biliary drainage could have multiple explanations. One explanation can be attributed to the biliary drainage or stent placement procedure itself. During this procedure, a gastroscope is inserted via the oral cavity and moved toward the duodenum. Once it is situated before the Papilla of Vater, a papillosfincterectomy is performed before the stent can be inserted [20]. This local incision might already lead to influx of duodenal bacteria into the pancreas, or perhaps the insertion of the stent might lead to migration of bacteria into the distal bile duct but also possibly into the pancreas itself. Another explanation might be the fact that the corpus alienum is left in the distal bile duct. Corpora aliena are notorious for providing a film in which bacteria can grow, for example synthetic heart valves that get infected with streptococci or staphylococci, or infections in joint replacement surgeries [21–25]. Long term intravenous antibiotic and sometimes even replacement of the infected material is needed to combat these infections. It is therefore not unlikely that the plastic or SEMS could become colonized after the barrier between the distal bile duct and duodenum has been destroyed. Swidsinski et al. studied the bacteria present on biliary stents by fluorescence in situ hybridization in diseased pancreatic and biliary tracts and showed that bacteria are able to form a biofilm on a distal bile duct stent [26]. These bacteria might migrate through the distal bile duct wall into the head of the pancreas. Corpora aliena can also induce swarming phenotypes of Proteobacteria, the most abundant phylum found in this study [27]. These swarming Proteobacteria are very mobile and are therefore able to translocate quickly.

We show that the pancreatic microbiome mostly consists of Proteobacteria, followed by FAFV and only contains a small load of Bacteroidetes. The twelve patients included in the analysis of Pushalkar et al., contained relatively more Bacteroidetes and only a small amount of FAFV [14]. However, these bacterial components were described in terms of relative abundance instead of actual, total abundance. When analyzing values of relative abundance in a sample in which almost no bacteria are present, the relative abundace value of a certain bacterial group can still be large if there are no other bacterial types present in the sample. In the samples of this current study with a low bacterial load, the results of Pushalkar et al. are more comparable to the results of this study. This suggest that perhaps the bacterial load in Pushalkar et al. might have been low, leading to relatively high percentages and large effects of small amounts of bacteria.

In the present study, not all patients had a bacterial load in the pancreatic biopsy. This is comparable with the study of Geller et al., who found bacterial DNA in 86 out of 113 patient samples and showed the relative abundance for 65 of those patients [7] Pushalkar et al., did however detect bacteria in all samples [14]. This might be due to the lower sample size, experimental methods introducing contamination, or clinical factors such as a biliary drainage, stent or PTCD placement, and PPI usage, all of which influence bacterial load. The increase in bacterial load in patients that use PPIs might be explained due to less acidic environment in the stomach. This prevents bacteria from dying, causing a higher residual load that can translocate from the duodenum to the pancreatic tissue. This is also shown by Imhann et al., who reported that the oral microbiome is more abundant in the gut microbiome of patients that use PPIs [28].

The increase in Proteobacteria due to biliary drainage might have a relevant clinical impact. As has been shown by Geller et al., Proteobacteria bacteria with expression of a long isoform of the bacterial enzyme cytidine deaminase are able to inactive the chemotherapeutic drug gemcitabine *in vitro* and *in vivo* [7]. This enzyme is seen primarily in Gammaproteobacteria such as *Klebsiella pneumoniae* and *E coli*. This could lead to a decrease in drug response and thus a decrease in disease free survival and overall survival. In this study, we did not see a statistically significant difference in Proteobacteria load between patients with short or long OS. However, only four patients with PDAC had a short OS. *Klebsiella* and *E*. *coli* were among the most abundant bacteria in the pancreatic microbiome in this study. In patients that did not undergo a biliary drainage, there was no abundance of *Klebsiella* and lower abundance of *E*. *coli* compared to patients that did undergo a biliary drainage. Unfortunately, there were not enough patients who received gemcitabine in this study to draw any conclusions regarding the effect on abundance of these bacteria and survival. When Geller et al. administrated the antibiotic drug ciprofloxacin to mice, gemcitabine became more effective and the tumors of the mice shrank [7]. Therefore, it might be likely that studying the microbiome after biliary drainage, and possibly altering the microbiome with antibiotics, could have great consequences for gemcitabine efficiency in patients with PDAC undergoing (neo)adjuvant chemotherapy treatment. A downside to the administration of antibiotics for *Klebsiella pneumoniae* is that it is known to correspond with an increase in resistance to chemotherapy and should therefore only be considered when more evidence is available. The effect of a single dose of prophylactic antibiotics during stent placement might also already have an effect on the bacterial load. However, it is not known how many bacteria translocate from the duodenum towards the stent when the stent is *in situ*.

Even though the patients included in this study already underwent treatment with preoperative diagnostics, drainage and antibiotics and underwent surgery, the obtained microbiome composition is representative for this group of patients peri- and postoperatively. If, as stated by Geller et al, the treatment outcomes are influences by the microbiome, knowledge of the postoperative microbiome composition is pivotal [7]. Furthermore, in a larger cohort study, knowledge of the perioperative microbiome composition might help predict complications. Due to the moment of sample collection, no statements can be made based on this study in regard to the influence of the microbiome on carcinogenesis.

Ideally, samples would be available of healthy pancreatic tissue and of treatment naïve pancreatic cancer patients. This could help understand the microbiome composition and bacterial load in the normal situation, and help study the direct effect of interventions such as biliary drainage. However, obtainment of pancreatic tissue in healthy patients or simultaneously during interventions such as biliary drainage is not desirable due to the high risk of complications and therefore no adequate control group is possible in human studies for the pancreatic microbiome.

In many cancer types such as colorectal cancer and cervical cancer, the microbiome has been linked to overall survival, as it has been in pancreatic cancer by Riquelme et al [29–31]. They showed that patients with shorter than 5-year survival, had a less diverse pancreatic microbiome compared to patients with over 10-year survival. In our study, we did not detect a statistical difference in diversity between short and long OS. This might be due to lack of statistical power, or that diversity only increases in long term survivors and that the tipping point might lie above one year. In our study, the follow up was not long enough to detect >5 year survivors. The median overall survival for pancreatic cancer is 18.1 months [32]. However, patients with more than a 10 year overall survival are rare and therefore examining smaller time differences might lead to a higher prognostic value for bacterial diversity and overall survival [33].

One of our goals was to find a substitute for the characterization of the pancreatic microbiome by the analysis of the duodenal microbiome. In this study, we have shown that samples from different duodenal sites are unsuitable as a substitute. Pushalkar et al. have compared the pancreatic microbiome to fecal samples and showed an increased relative abundance of Proteobacteria in the pancreatic microbiome compared to the gut [14]. We also found an increased relative abundance of Proteobacteria in pancreatic tissue compared to duodenal fluid, but not to the other duodenal materials. These data suggest that the microenvironment of the pancreas is more favorable for the outgrowth of Proteobacteria compared to the other phyla. This might be due to the presence of bile flowing though the stent. Bile is known to be bactericidal, and *E*. *coli*, *Klebsiella* and *Enterococcus* species are resistant to this [34].

Also between the duodenal materials, there was a difference in diversity and in the correlation among each other. From the colon microbiome, it is known that the adherent bacteria that are found when taken a tissue biopsy differ from the luminal bacteria in the fecal microbiome [35]. This also appears to be the case for the duodenal microbiome, causing them to have a weak to moderate correlation. The duodenal swab has the highest bacterial load and diversity, which is also suggestive for the two different micro milieus in the duodenum as there is still some duodenal fluid present on the papilla of Vater and the swab is taken from the tissue. Another additional explanation for the differences found between the duodenal microbiomes, might be due to the slightly different bacterial DNA isolation protocols. Due to different compositions of the fluids and since the tissue first had to be broken down, additional steps and different quantities of lysis buffer were required.

This study has several limitations. Patients with different diseases and clinical demographics were included, leading to small subsets for some specific clinical variables. This has prevented multivariate analyses. However, to correct for major influences such as biliary drainage and PDAC, subgroup analysis were performed. Furthermore, follow-up of some patients was less than one year, leading to exclusion of those patients for survival analysis. Additionally, as these samples were collected in a biobank, no negative controls were taken along at time of sample collection. However, the very low bacterial load in samples without biliary drainage suggests that there is very limited to no contamination. Moreover, samples were only obtained at the time of operation. Consequently, analysis of the change in microbiome before and after biliary drainage and/or PTCD or stent was not possible. Finally, as this is an explorative study with a relative small sample size, no correction for multiple comparisons was performed. This would lead to a high type II error, which is not desirable for explorative studies [36].

Therefore, future research is needed to confirm our finding in a larger cohort and to study the direct effect of biliary drainage and stent or PTCD placement in the distal bile duct, by comparing the microbiome before stent placement and during surgery. When this is performed in a larger cohort, this might also help to better understand its effect on (neo)adjuvant treatment and survival in patient suffering periampullary and pancreatic malignancies, including PDAC.

In conclusion, we show that the bacterial load within the pancreas is not only higher peroperatively in patients that underwent biliary drainage, but also differs at phylum and species level. Furthermore, PPI usage and the presence of PDAC correlates with a higher microbial load. The increased microbial load might influence survival, complications and the efficiency of (neo)adjuvant therapy and should therefore be further investigated.

## Supporting information

S1 Fig Relative abundance. This figure presents the relative abundance of Proteobacteria, FAFV and Bacteriodetes for all paired samples.
(PDF)

S1 File Statical outcomes comparisons bacterial load. All p values of performed comparisons regarding abundance, relative abundance and diversity are provided here.
(XLSX)

S2 File Raw data of all samples. Here raw data for all analyzed samples is provided. On the first tab, the load per sample is provided for each phyla. On the second tab, each line represents a specific fragment length for a specific sample for a particular primer set with the corresponding intensity of that fragment.
(XLSX)
