# Supplementary figures and images for: Factors that influence the pancreatic and duodenal microbiome in patients undergoing pancreatic surgery
Source: PLoS One. 2022 Dec 16;17(12):e0278377. doi: 10.1371/journal.pone.0278377 (PMC9757549; doi:10.1371/journal.pone.0278377)

Relative abundance of paired samples

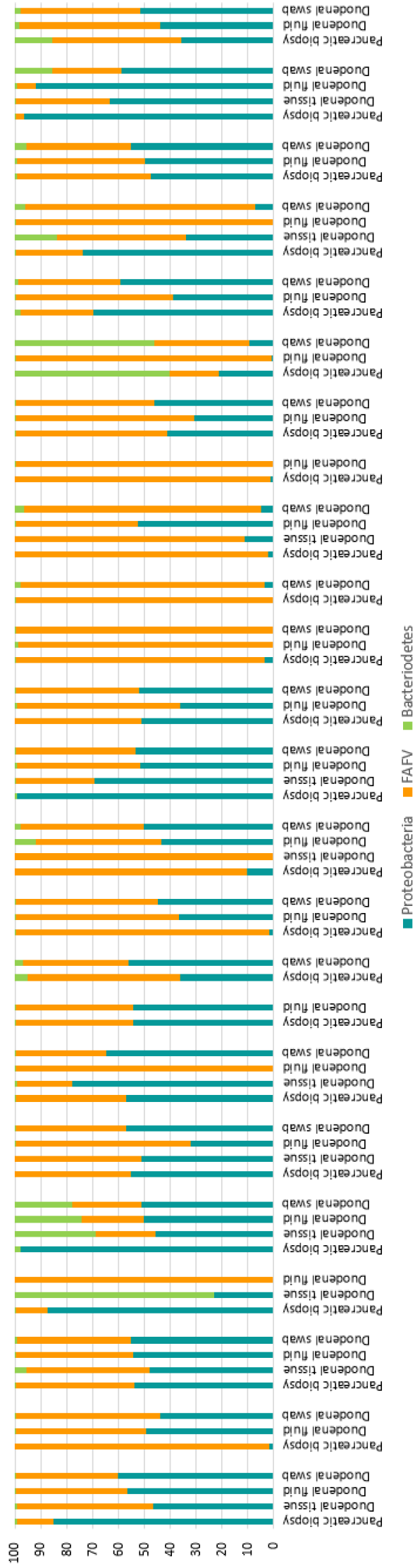

Supplement: S1 Fig — This figure presents the relative abundance of Proteobacteria, FAFV and Bacteriodetes for all paired samples. (PDF) [file pone.0278377.s001.pdf]
